# Corpus Callosum Infarct in the Background of Varicella-Zoster Infection: A Report of a Rare Case

**DOI:** 10.7759/cureus.29943

**Published:** 2022-10-05

**Authors:** Hira Farooq, Alishbah Ziad, Quratulain Khan, Anis Rehman, Kashif Siddique

**Affiliations:** 1 Radiology, Shaukat Khanum Memorial Cancer Hospital and Research Centre, Lahore, PAK

**Keywords:** aspergillus, infection, varicella zoster, acute lymphoblastic leukemia, corpus callosum infarct

## Abstract

Infarctions of the corpus callosum are rare due to a rich blood supply. Corpus callosum derives its blood supply from four vessels from the anterior and posterior circulation and for this reason, they have a rare, atypical presentation. There is scarce literature regarding this pathology. Corpus callosum infarcts usually present with non-specific signs and symptoms. Here, we describe a case of corpus callosum infarction in a 5-year-old boy who was a known case of acute lymphoblastic leukaemia. He presented with disseminated varicella infection and developed tonic-clonic seizures. MRI brain was performed and a diagnosis of corpus callosum infarct was made. The patient was treated conservatively.

## Introduction

Corpus callosum makes the largest association fibre of white matter [1]. However, owing to its rich and extensive blood supply from branching vessels of both the anterior and posterior circulation, these infarcts are not encountered commonly and are extraordinary and infrequently documented in the literature. Corpus callosum infarcts present with slow developing and generalized, non-specific signs and symptoms [1, 2]. For this reason, they are often misdiagnosed [3].

Corpus callosum functions as a sensory and motor pathway as well as a communication between the left and right cerebral hemispheres [4]. Infarcts in this part of the brain can occur in the background of varicella-zoster encephalitis. Varicella-zoster virus can lead to cerebrovascular events of both intracranial and extracranial arterial circulations, more commonly in the intracranial arteries [5]. Patients with varicella-zoster encephalitis may present with prolonged headaches and have developed secondary to ischemic or hemorrhagic strokes, carotid artery dissection or aneurysmal subarachnoid haemorrhage [5].

## Case presentation

We present the case of a 5-year-old male child who was a known case of pre-B-cell acute lymphoblastic leukaemia (ALL) and was under treatment in our hospital. The patient was undergoing chemotherapy for the known primary diagnosis. The treatment protocol was UKALL interim Guidelines Regimen B (vincristine sulphate IV 1.02 mg, sodium chloride 0.9% infusion 25 mg, methotrexate injection 12 mg). He had a history of angioinvasive aspergillus with invasion into the hard palate in 2020, for which he had received antifungal therapy and debridement of fungal sinuses disease. 

The patient had direct contact with chickenpox through siblings and developed chicken pox in January 2022. Polymerase chain reaction (PCR) for herpes simplex virus (HSV)-1, HSV2, and varicella-zoster virus (real-time) was performed, which turned out to be positive for varicella-zoster virus. The results of the real-time PCR (blood specimen) is given in Table 1.

**Table 1:** Positive PCR (real-time) for varicella-zoster virus HSV: herpes simplex virus; VZV: varicella-zoster virus; PCR: polymerase chain reaction

TEST	RESULTS/UNITS
HSV1	0 copies/mL
HSV1 Result	Not detected
HSV2	0 copies/mL
HSV2 Result	Not detected
VZV	14400 copies/mL
VZV Result	Detected

**Figure 1:**
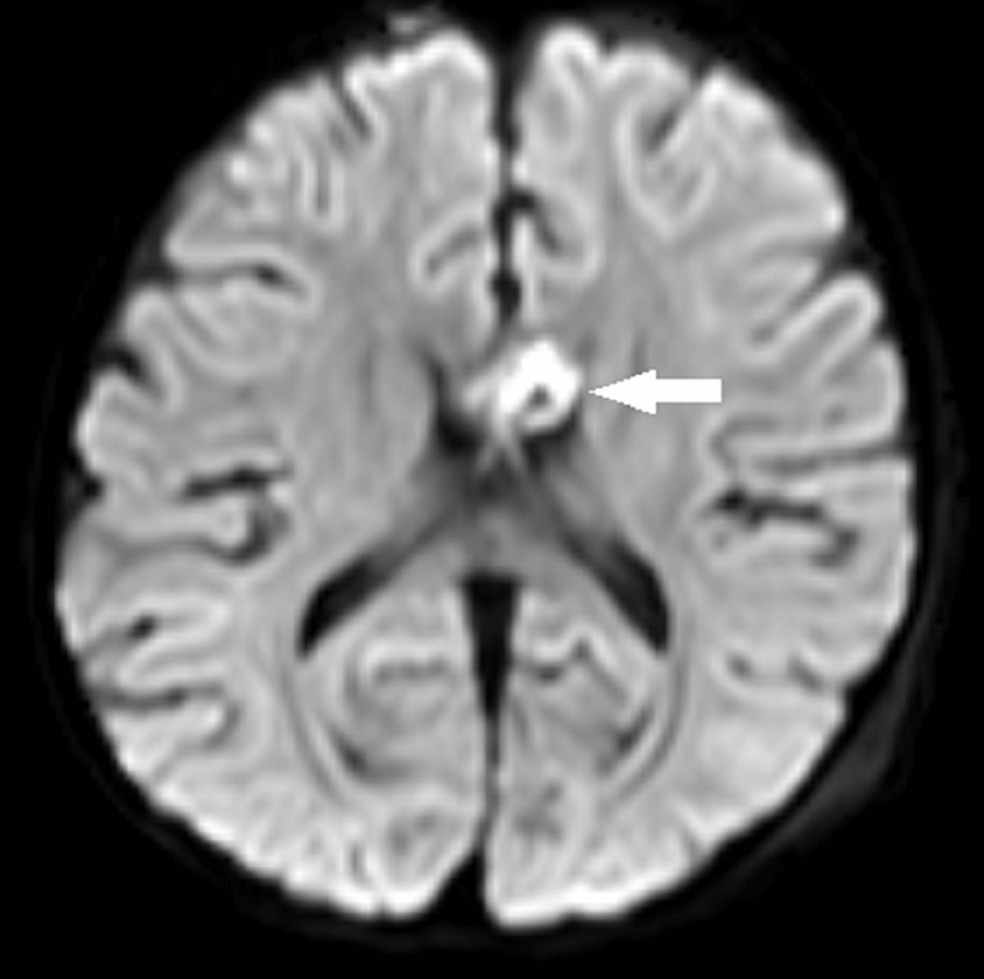
Axial MRI brain DWI image showing focus of hyperintense signal involving the genu of corpus callosum (white arrow) consistent with infarct DWI: diffusion-weighted imaging

**Figure 2:**
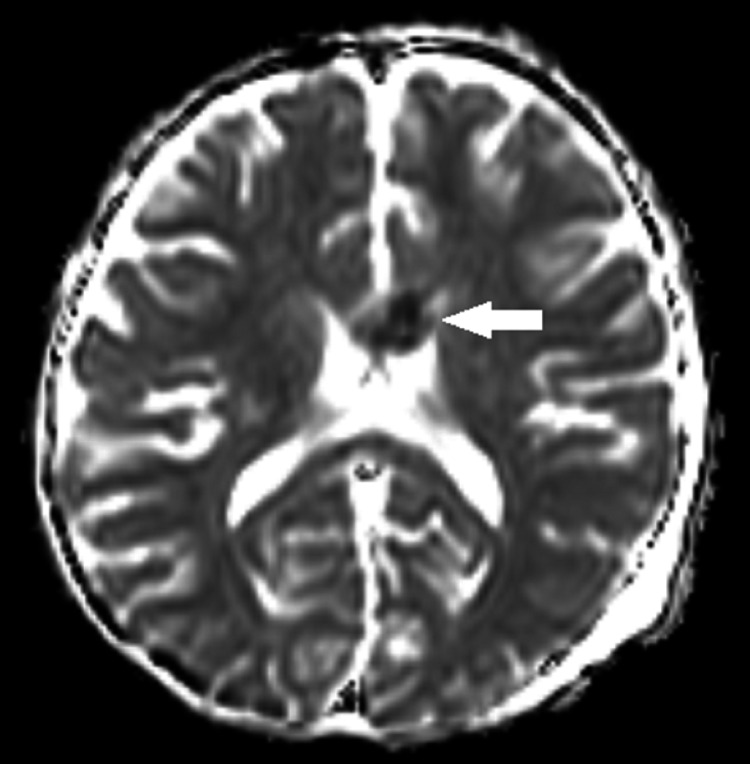
Axial MRI brain ADC image showing hypointense signal (white arrow) involving the genu of corpus callosum (corresponding to high signal on DWI) consistent with infarct ADC: apparent diffusion coefficient; DWI: diffusion-weighted imaging

The patient remained in the ICU due to chicken pox pneumonia. During his ICU stay, he developed tonic-clonic seizures, for which MRI was performed. MRI showed multifocal infarcts involving the genu and body of corpus callosum and internal capsules bilaterally. The MRI brain axial diffusion-weighted imaging (DWI)/apparent diffusion coefficient (ADC) images (Figures 1, 2) depicted hyperintense signal involving the genu of corpus callosum on DWI image with corresponding hypo intense signal on ADC mapping consistent with infarction.

**Figure 3:**
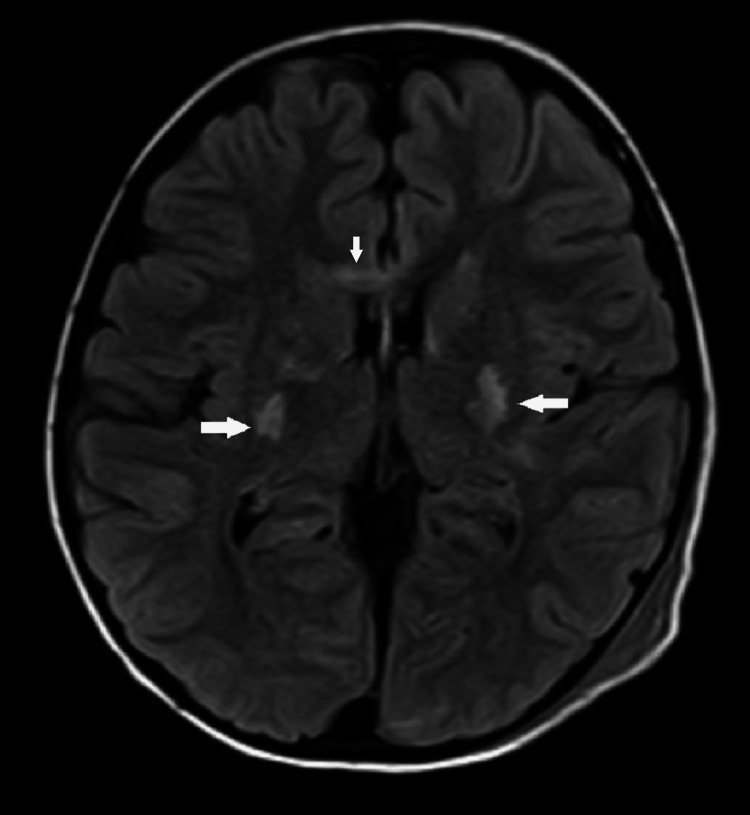
MRI brain axial FLAIR images depicting symmetrical hyperintensities (white arrows) involving bilateral basal ganglia and genu of corpus callosum FLAIR: fluid-attenuated inversion recovery

**Figure 4:**
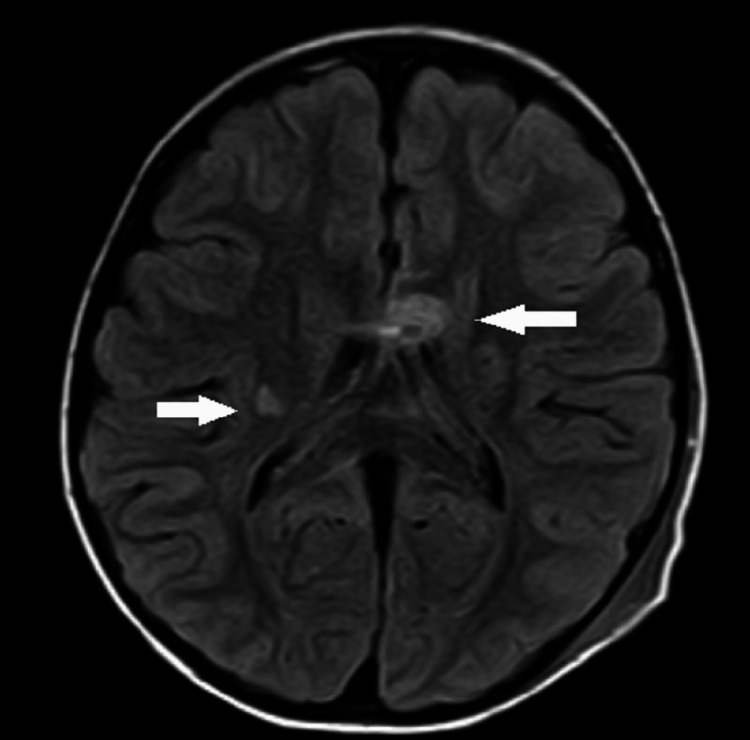
MRI brain axial FLAIR images depicting hyperintense foci involving the right-sided basal ganglia and genu of corpus callosum (white arrows) FLAIR: fluid-attenuated inversion recovery

The MRI brain axial fluid-attenuated inversion recovery (FLAIR) sequences are shown in Figures 3, 4, depicting symmetrical hyperintense foci involving bilateral basal ganglia and genu of corpus callosum.

**Figure 5:**
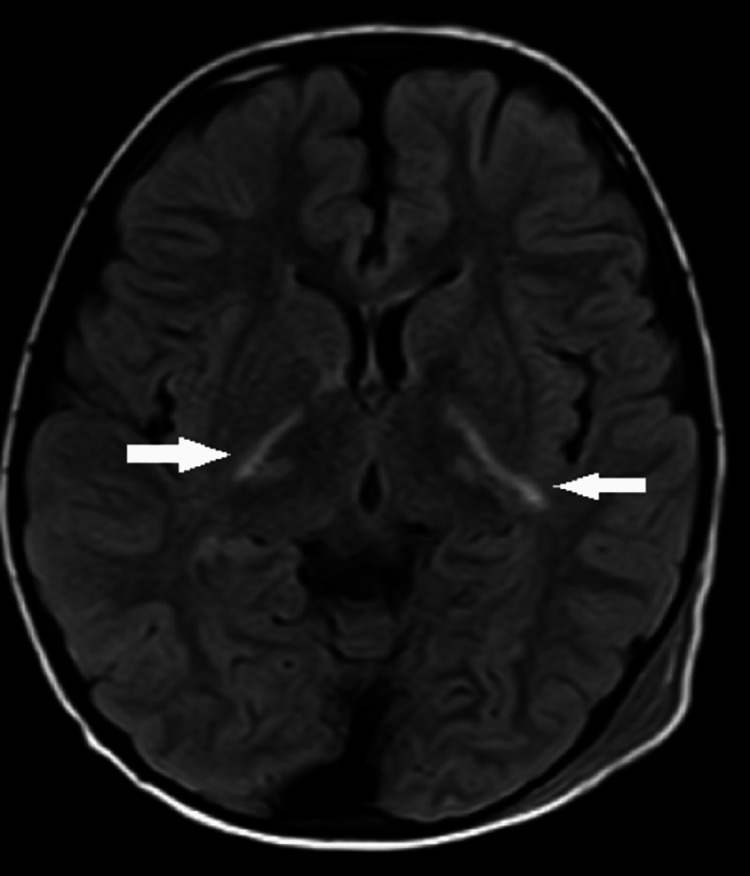
MRI brain axial FLAIR images depicting hyperintensities involving bilateral posterior limbs of internal capsule FLAIR: fluid-attenuated inversion recovery

MRI brain axial FLAIR sequence is shown in Figure 5, demonstrating symmetrical hyperintense signal involving posterior limbs of bilateral internal capsules. 

**Figure 6:**
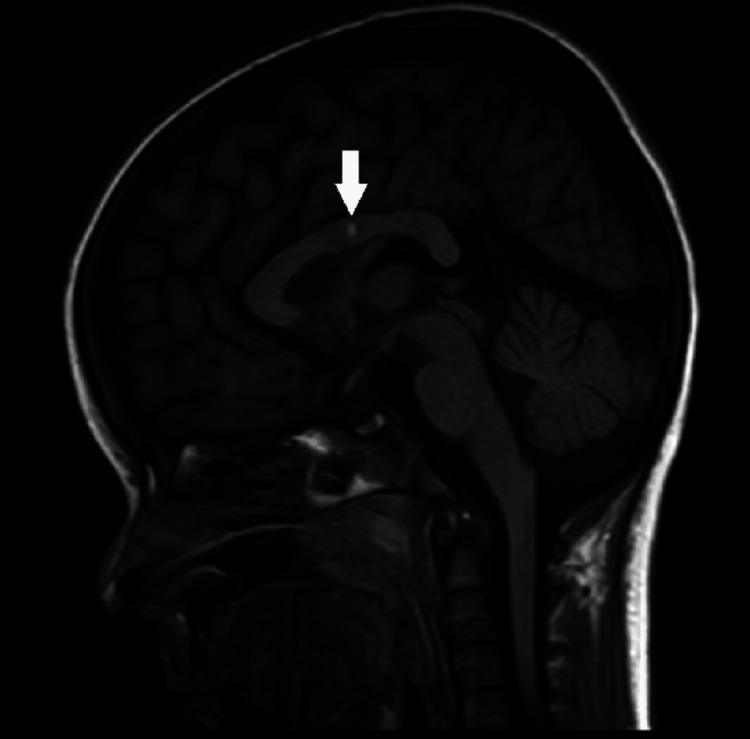
MRI brain non-contrast T1WI sagittal reformatted sequence demonstrating focus of T1 hyperintensity in the body of corpus callosum (white arrow) suggestive of hemorrhagic nature of infarct of corpus callosum

Figure 6 shows a non-contrast T1WI sagittal reformatted sequence showing hyperintense focus in the body of corpus callosum suggestive of the hemorrhagic nature of infarct.

The patient was treated in an ICU setting with acyclovir (280 mg three times a day) for four weeks. He gradually recovered until asymptomatic and was discharged from ICU.

## Discussion

The corpus callosum has a rich vascular supply by branching arteries from both the anterior and posterior circulation, comprising anterior and posterior peri callosal arteries (branches of anterior and posterior cerebral artery, respectively). Further, the corpus callosum is also supplied by branches from the anterior communicating arteries (subcallosal and medial callosal arteries) [6]. Therefore, blood circulation of the corpus callosum is rarely blocked and infarcts of the corpus callosum are less likely.

Rich and extensive collateralization of the anterior and posterior circulation supplying the corpus callosum results in protective redundancy and resistance of the posterior peri callosal arteries to occlusion by the emboli due to its right angle formation with its parent vessels, resulting in the rarity of corpus callosum infarcts. However, background encephalitis resulting in multifocal vasculopathy as in this presented case can result in the patient developing this rare entity. Further, background malignancy resulting in an immunocompromised state of the patient poses an additional threat. Other than encephalitis, various causes of corpus callosum infarction include atherosclerosis, embolization, vasospasm, hypoxia, and hyper coagulopathies.

The presenting symptoms of acute corpus callosum infarction are non-specific. These may include headaches, limb weakness, movement disorders, and memory impairment [4]. Ischemic infarction of the corpus callosum is most commonly known to occur in the splenium. Infarcts in splenium are often associated with bilateral cerebral hemispheric involvement in about 46.2% of the patients according to a recent study. Whereas atherosclerosis is commonly associated with genu and/or body of corpus callosum infarcts [4].

The most common cause of viral encephalitis in immunocompetent and immunocompromised patients include infection from HSV-1, varicella-zoster virus, and Epstein-Barr virus [7]. Vasculopathy caused by the varicella-zoster virus can be both unifocal or multifocal and affect both immunocompetent and immunocompromised patients [8]. In immunocompromised patients, varicella-zoster virus vasculopathy may involve small vessels, especially in those patients who have underlying malignancy or acquired immune deficiency syndrome [8,9]. As in our case, the patient had underlying malignancy (ALL). Varicella-zoster virus vasculitis unifocal involvement is mostly observed in elderly immunocompetent patients whereas, in immunocompromised patients, varicella-zoster virus infection affects both large and small arteries resulting in multifocal vasculopathy [10].

Treatment options for varicella-zoster virus-induced vasculopathy are mostly empirical due to the lack of randomized controlled trials. Empirical therapy generally includes the drug acyclovir, given either in combination with steroids or without steroids. However late diagnosis or misdiagnosis because of non-specific and atypical presentation of the disease results in only around 60% cure rate of patients diagnosed with varicella-zoster virus vasculitis. The duration of treatment is, however, variable and depends on the patient’s clinical condition [11].

## Conclusions

Varicella-zoster virus vasculitis is an important and treatable cause of vasculopathy on the background of encephalitis. Especially in immunocompromised patients, varicella-zoster encephalitis results in multifocal vasculopathy that needs to be diagnosed and treated properly and timely. Correlation between clinical presentation and imaging findings is required for timely diagnosis. Since the rarity of corpus callosum infarcts poses a diagnostic dilemma, timely radiological imaging and diagnosis would be helpful in high-risk patients. Particularly, in patients with varicella encephalitis, hemorrhagic corpus callosum infarcts can be managed better with prompt diagnosis.
